# Lamivudine monotherapy as a safe option for HIV-infected paediatric clients with adherence challenges: new evidence from a large South African cohort

**DOI:** 10.7448/IAS.17.4.19763

**Published:** 2014-11-02

**Authors:** Verena Linder, Cheree Goldswain, Gerald Boon, Craig Carty, Valerie Jackson, Kim Harper, John Lambert

**Affiliations:** 1 Health, Paediatrics, Eastern Cape, East London, South Africa; 2 Social and Clinical Research, The Relevance Network, Johannesburg, South Africa; 3 Clinical Audits and Surveillance, The Rotunda Hospital, Dublin, Ireland; 4 Infectious Diseases, Mater Misericordiae University Hospital, Dublin, Ireland

## Abstract

**Introduction:**

HIV-infected children in resource-poor settings comprise a unique population who require antiretroviral therapy (ART) in careful consideration of social and structural barriers to compliance. Given these aggregate challenges and emerging research into “holding” treatment options, we investigated the efficacy of lamivudine monotherapy (LM) as an alternative to more complex second and third line therapies.

**Methods:**

A retrospective review of all eligible LM events (=6 months) from a cohort of two linked health facilities in the Eastern Cape Province, South Africa was undertaken. Events were disaggregated according to absolute CD4 count at initiation (Group 1: >200 cells/L, n=64; Group 2:=200cells/L, n=10). Study endpoints were defined as a decline of absolute CD4=200 cells/L (Group 1), WHO stage 3 or 4 event (Groups 1& 2), or initiation of second or third line (Groups 1 & 2).

**Results:**

Seventy-four eligible LM events were identified among 71 HIV-positive children (58% male; median age at LM 9.7 years and median LM duration 11.5 months). CD4 decreases and measured WHO stage 3 or 4 events did not yield overall significance between groups (Table 1). No deaths were recorded.

**Conclusions:**

LM offers a promising alternative approach to ART management in young patients with an absolute CD4 >200 cells/L pending availability and/or willingness to adhere to second or third line therapies. In more immunocompromised children, LM may be considered as a last option if either the child or caretaker has concerns about second or third line management, or has defaulted repeatedly.

**Table 1:** Characteristics for LM-initiated patients

	All LM Events (n=74)	Group 1: Baseline CD4 200 cell/µL (n=64)	Group 2: Baseline CD4=200 cells/µL (n=10)	p
Male (n, %)	43 (58)	38 (59)	5 (50)	0.576
Age at LM initiation, years (median, IQR)	9.7 (6.5–11.8)	9.0 (6.4–11.2)	11.9 (10–14.4)	0.010
Duration of LM, months (median, IQR)	11.5 (7.1–17.4)	12.9 (7.4–17.9)	7.1 (5.1–9.1)	0.126
Final CD4, cells/µL (IQR, final prior to switch or last on LM)	429 (212–593)	471 (325–632)	29 (16–59)	0.0001
Patients with overall decrease in CD4 (n, %)	64 (86.5)	58 (90.6)	6 (60)	0.185
Patients with 25% decrease in CD4 (n, %)	50 (67.6)	44 (68.8)	6 (60)	0.325
Patients switched to second or third line therapy (n, %)	17 (23)	11 (17.2)	6 (60)	0.003
Stage 3 or 4 event (n, %)	6 (8.1)	5 (7.8)	1 (10)	0.814

